# Tsetse peritrophic matrix influences for trypanosome transmission

**DOI:** 10.1016/j.jinsphys.2019.103919

**Published:** 2019-10

**Authors:** Serap Aksoy

**Affiliations:** Department of Epidemiology of Microbial Diseases, Yale School of Public Health, 60 College St, LEPH 624, New Haven, CT 06520, United States

## Abstract

Tsetse flies are important vectors of parasitic African trypanosomes, agents of human and animal trypanosomiasis. Easily administrable and effective tools for disease control in the mammalian host are still lacking but reduction of the tsetse vector populations can reduce disease. An alternative approach is to reduce the transmission of trypanosomes in the tsetse vector. The gut peritrophic matrix (PM) has emerged as an important regulator of parasite transmission success in tsetse. Tsetse has a Type II PM that is constitutively produced by cells in the cardia organ. Tsetse PM lines the entire gut and functions as an immunological barrier to prevent the gut epithelia from responding to commensal environmental microbes present in the gut lumen. Tsetse PM also functions as a physical barrier to trypanosome infections that enter into the gut lumen in an infective blood meal. For persistence in the gut, African trypanosomes have developed an adaptive manipulative process to transiently reduce PM efficacy. The process is mediated by mammalian trypanosome surface coat proteins, Variant Surface Glycoproteins (VSGs) which are shed in the gut lumen and taken up by cardia cells. The mechanism of PM reduction involves a tsetse microRNA (miR-275) which acts thru the Wnt signaling pathway. The PM efficacy is once again reduced later in the infection process to enable the gut established parasites to reenter into the gut lumen to colonize the salivary glands, an essential process for transmission. The ability to modulate PM integrity can lead to innovative approaches to reduce disease transmission.

## Highlights

• Tsetse PM is constitutively produced by cardia and lines the entire gut.
• PM prevents epithelial immune induction against resident microbiota.
• PM is a physical barrier trypanosomes must bypass for transmission.
• Trypanosomes modify tsetse *microRNA-275* in cardia to reduce PM synthesis.
• Obligate symbiont *Wigglesworthia* influence PM development in larva.

## Graphical abstract

**Figure:**
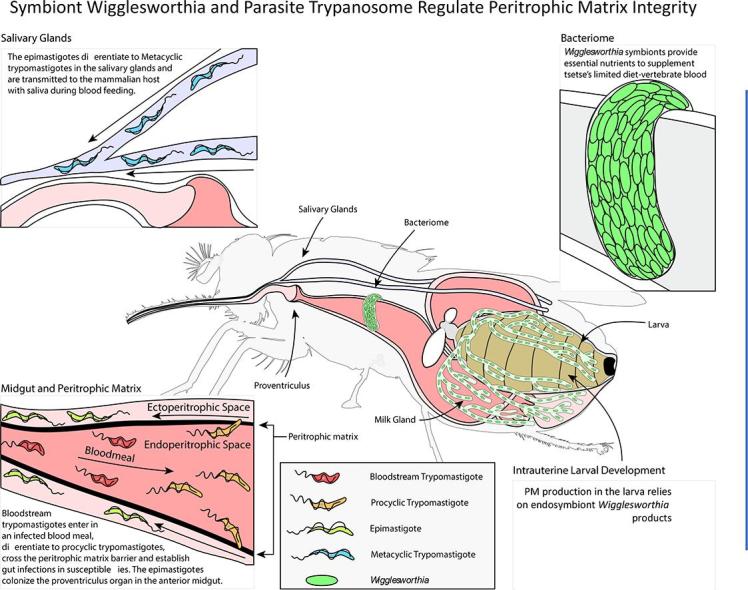

African trypanosomiasis devastates human and animal health in sub-Saharan Africa. Tsetse are the sole vectors of the disease agents, protozoan African trypanosomes. Overall, 60 million people live in disease-endemic regions in Africa, and cases have numbered in the hundreds of thousands during epidemic periods (Ekwanzala, 1996, Barrett, 1999, Abel, 2004, Jannin, 2005, Barrett, 2006). An ambitious campaign led by WHO and international partners has recently dramatically reduced the prevalence of human African trypanosomiasis (HAT) through active surveillance and treatment of patients (WHO, 2014, Franco et al., 2014), particularly in west-Africa where disease relies on human-fly transmission cycle. Only recently, a new drug, fexinidazole, has been approved for use to cure within 10 days Gambiense form of the disease that occurs in West and Central Africa (Chappuis, 2018). Control of the Rhodesiense form of the disease that impacts East Africa where disease transmission also involves animal reservoirs is more difficult and requires vector control applications. For optimal disease prevention, vaccines and diagnostic assays applicable in the field are still lacking. Practical interventions (Lehane, 2016, Solano et al., 2013, Courtin, 2015) and empirical models (Rock et al., 2015, Gilbert, 2016, Davis et al., 2011) suggest that vector control is essential for sustainable HAT elimination in the foreseeable future. In addition to Trypanosoma brucei that causes HAT, related parasites cause a chronic wasting disease in domesticated livestock, known as Animal African Trypanosomiasis (AAT) or nagana. Animal diseases inflict devestating economic losses throughout subSaharan Africa. Hence, effective and cheap methods targeting tsetse viability, or parasite transmission through tsetse vector are desirable and can expand the tool box available for the control of these diseases.

Trypanosome transmission in tsetse is a dynamic process in which parasites encounter several barriers that restrict their transmission potential (Aksoy et al., 2003, Dyer et al., 2013, Matthews et al., 2015) (Fig. 1). Infections in the gut begin when mammalian bloodstream form parasites (BSF) are acquired via an infectious blood meal. Among the important factors that affect tsetse’s parasite transmission ability (known as vector competence) are epithelial immune responses (Hao, 2001, Hu and Aksoy, 2005, MacLeod et al., 2007, Wang and Aksoy, 2012, Haines et al., 2010) that include trypanocidal antimicrobial peptides (Hao, 2001, Hu and Aksoy, 2005), reactive oxygen intermediates (ROIs) (MacLeod et al., 2007), PGRP-LB (Wang and Aksoy, 2012) and tsetse-EP protein (Haines et al., 2010). In addition, both tsetse’s microbial fauna (Michalkova et al., 2014, Bing, 1857, Wang et al., 2013, Weiss and Aksoy, 2011, Weiss et al., 2012, Weiss et al., 2011, Weiss et al., 2013) and a parasite-mediated host manipulative process can influence the integrity of critical immune barriers, such as the gut Peritrophic Matrix (PM) structure, to favor parasite survival (Aksoy et al., 2003, Wang and Aksoy, 2012, Weiss et al., 2011, Weiss et al., 2013, Weiss et al., 2014, Aksoy et al., 2014, Alam, 2012, Hao et al., 2001, Hu and Aksoy, 2006, Vigneron, 2018, Wang et al., 2009, Wang, 2008). Here, we will focus on the role of the PM structure for gut parasite infection establishment, on the molecular aspects of the tsetse-trypanosome dialogue that modulate PM integrity to favor parasite transmission success and on role of the microbiota for PM development. Knowledge on the tsetse-trypanosome dialogue and manipulation of PM integrity by parasites has the potential to advance development of innovative methods to block parasite transmission in tsetse as an alternative biological approach to control disease.

**Fig. 1:**
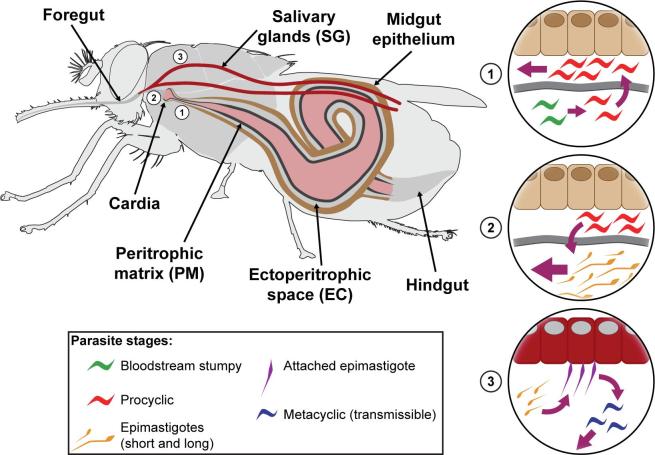
Trypanosome transmission through tsetse.

## 1 Role of PM in gut pathogen colonization

The PM, which is present in the midgut of most insects, regulates digestive processes by passively controlling the movement of digestive enzymes into the gut lumen, protects midgut epithelial cells from environmental toxins and mechanical damage caused by ingested food particles, and prevents or reduces the severity of pathogen infections (Hegedus et al., 2009, Abraham, 2004, Lehane, 1997). While adult Diptera, such as mosquitoes and sandflies, have Type I PMs that are produced by midgut epithelial cells upon blood feeding, adult tsetse flies have a Type II PM that is constitutively produced, regardless of feeding status, by cells in the fly’s cardia (also called proventriculus - a distinct tissue that defines the foregut-midgut junction). Tsetse’s PM is composed of three chitinous layers interspersed with glycosaminoglycans and glycoproteins (Lehane et al., 1996) and is comparable in structure to a sleeve that lines the gut lumen. It has been shown that newly eclosed tsetse adults (referred to as “teneral”) lack a strong PM when they first emerge from their pupal case. However, formation of the PM structure is complete after acquiring one or two blood meals within the first 90 h of adulthood (Lehane and Msangi, 1991). Interestingly, teneral tsetse are highly susceptible to infection when they are provided with trypanosomes in their first adult blood meal, while mature (1 week old) adults that have received several blood meals are resistant to gut parasite colonization (Welburn and Maudlin, 1992, Haines, 2013). The structural integrity of tsetse’s PM increases as a function of adult age post-pupal eclosion, and the higher parasite susceptibility presented by young adults has been attributed to the absence of a robust PM at this stage of host development.

Variable functional relationships exist between the PM and pathogen infection outcomes in different insects. For example, following challenge with *Plasmodium* parasites, *Aedes aegypti* that have an artificially thickened PM harbor fewer oocysts than their counterparts with a normal PM (Billingsley and Rudin, 1992). Similarly, *Plasmodium* oocyst formation was completely blocked in the midgut of *A. aegypti* when the thickness of the mosquito PM was increased by adding a chitinase inhibitor (allosamidin) to their diet (Shahabuddin et al., 1993, Shahabuddin et al., 1995). When the expression of *A. aegypti chitin synthase* was knocked down through the use of RNAi, treated flies had fewer *Plasmodium* oocysts than did their wild-type counterparts (Kato, 2008). In sand flies, in one study the PM was shown to block parasite development and colonization of the gut (Coutinho-Abreu et al., 2013). In another study, sandflies that presented a structurally compromised PM were shown to be less susceptible to midgut *Leishmania* infections (Pimenta et al., 1997). It is suggested that in sand flies, the presence of PM may create a barrier to prevent the rapid diffusion of digestive enzymes that are otherwise damaging to parasites during the early infection period when they undergo differentiation and are vulnerable to proteolytic damage (Shahabuddin et al., 1995). In ticks, intact PM was found to be essential for efficient gut colonization by the Lyme disease agent, *Borrelia burgdorferi* (Narasimhan, 2014). Interestingly, the PM integrity of the tick vector was found to be regulated by the gut microbiota, which influenced PM associated gene expression through the regulation of a critical transcription factor (Kato, 2008). Collectively, findings from multiple vector-pathogen systems suggest that the PM may influence pathogen colonization through different mechanisms. Presence of the PM may impact the passage of digestive enzymes with chemical properties that influence the differentiation or colonization of parasites. Alternatively, PM may present a physical block which pathogens have to bypass for colonization by secreting specialized enzymes. Finally, the gut microbiota may also regulate PM integrity, which in turn influences vector pathogen interactions and infection outcome.

To understand the role of PM for immune surveillance in tsetse, we used RNA interference-based reverse genetics to inhibit the production of a structurally robust PM in tsetse by targeting the major PM glycoproteins as well as *chitin synthase.* We next evaluated how the reduced PM integrity impacted infection outcomes after *per os* challenge with entomopathogenic exogenous bacteria (*Enterobacter sp*. and *Serratia marcescens* strain Db11). Entomopathogen proliferation was impeded in the RNAi treatment group with reduced PM integrity and flies survived significantly longer than the wild-type untreated controls. This was because the RNAi treatment group with reduced PM integrity could detect the presence of pathogens in the gut lumen and express antimicrobial peptides (AMPs) that could clear the pathogenic infection earlier in the process than did their counterparts with a fully developed PM. Hence, tsetse PM could serve as an immunological barrier that influences the fly's ability to detect and respond to the presence of exogenous microbes (Weiss et al., 2014). The presence of a strong PM may prevent unnecessary immune activation to the environmentally acquired commensal microbes present in the gut lumen.

We next evaluated the role of PM in trypanosome transmission outcome in tsetse using the same approach for experimental reduction of the PM. During their transmission cycle, trypanosomes colonize tsetse’s midgut, traversing the PM twice: first when they enter tsetse’s ectoperitrophic space (ES, area between the PM and gut epithelia) following ingestion, and second when they move back into the anterior gut region before migrating into the fly’s salivary glands (SG) (Shown in Fig. 1) (Van den Abbeele et al., 1999). After challenge with trypanosomes, RNAi treatment group with reduced PM integrity was more susceptible to gut parasite infection establishment implicating tsetse's PM as a physical impediment to parasite survival early in the infection process. We noted that once parasites bypassed the PM barrier however, they were eliminated by tsetse’s epithelial immune responses, including AMPs and reactive-oxygen intermediates (ROIs) (Vigneron, 2018). We noted that a subset of flies remained colonized by parasites in the midgut and further about 50% of these gut infected flies gave rise to SG infections that could be transmitted to the next mammal. In flies with only midgut infections, parasites remained in the ES of the gut and failed to cross the PM barrier in the cardia to enter into the lumen. However, in flies with midgut and SG infections, parasites could bypass the PM and enter into the gut lumen and then invade the SG through the mouthparts (Vigneron, 2018). In flies with MG and SG infections, the expression of genes encoding components of the PM were reduced in the cardia, and structural integrity of the PM barrier was compromised. Furthermore, we were able to increase SG infection prevalence by experimental reduction of PM through silencing of critical PM associated genes later in the infection process. Collectively, PM appears to be a barrier to trypanosome infection success both early in the infection process when parasites cross into the ES, and later when parasites have to escape from the ES to colonize the SGs.

## 2 Trypanosome manipulation of PM integrity for transmission success

We next investigated the molecular mechanisms by which the tsetse PM is compromised at critical times to further parasite transmission success. Trypanosome infections in the tsetse gut begin with mammalian bloodstream form parasites (BSF) that are acquired via an infectious blood meal. Two forms of BSF parasites, termed slender and stumpy, are present in the vertebrate blood (Matthews et al., 2015). Slender forms proliferate and cause the devastating effects of disease in vertebrates. Stumpy forms, which are developmentally-arrested, accumulate at high parasitemia and are responsible for continuing the disease cycle in tsetse. Upon entering the tsetse gut lumen, slender forms are readily lysed, releasing their cellular components into the gut environment. In contrast, stumpy forms, which are pre-adapted for survival in tsetse, differentiate to insect-stage procyclic form (PCF) within hours (Turner et al., 1988, Aksoy, 2016) and bypass the PM barrier and begin to replicate in the ES (Gibson and Bailey, 2003).

Transcriptomic analysis of midgut tissue 24, 48 and 72 h post parasite acquisition revealed significant reduction of expression in genes encoding PM associated products (Aksoy, 2016). We hypothesized that a parasite-derived manipulative process could transiently compromise tsetse’s PM integrity early in the infection process to facilitate parasite traversal of this barrier. We observed that the thick GPI-anchor containing parasite surface coat antigens, Variant Surface Glycoprotein (VSG), which are shed in the gut lumen early during differentiation to PCF, are taken up by the cardia organ responsible for synthesizing the major PM associated products. VSG uptake transiently reduced expression of genes that encode PM associated proteins, thus reducing the structure’s integrity temporarily and enabling the parasites to bypass the PM to colonize the ES of the gut (Aksoy, 2016). Administration of purified soluble VSG (sVSG, GPI hydrolyzed) also enhanced MG infection prevalence, further validating the role of VSG in the PM erosion process (Aksoy, 2016). Similar transcriptomic analysis of small RNAs have shown that expression of a tsetse microRNA (*miR-*275) is significantly reduced in the cardia at this time (Aksoy, 2016). Both VSG exposure and experimental silencing of *miR-275* in tsetse dramatically reduce the expression of PM-associated *peritrophins* (*pro1-3*), as well as the Iroquois/IRX family of protein-encoding genes and the extracellular ligand *wg,* suggesting that VSG acts through the Wnt signaling pathway*.* The molecular targets of *miR-275* and the regulation of *miR-275* expression remain to be investigated during parasite colonization.

To understand the role of PM later in the infection process, we similarly performed transcriptomic analysis of the midgut organ from flies that have MG parasite infections only and from flies that have both MG and SG infections as described earlier. We noted that PM associated gene expression in the cardia was only reduced in the flies with MG and SG infections. Our findings suggest that a parasite mediated manipulative process may again enable the gut established parasites to bypass PM barrier later in the infection in route to SG organ for transmission (Vigneron, 2018). The midgut parasite components that may mediate this PM manipulative process later in the infection process remains to be discovered.

## 3 Role of tsetse’s microbiota in PM development in juvenile stages

Tsetse has established an ancient obligate symbiosis with the bacterium, *Wigglesworthia glossinidia. Wigglesworthia* symbiosis impacts tsetse’s nutritional, reproductive and immune physiologies and also contributes to PM development, especially during the juvenile stages. Both female and male tsetse feed exclusively on nutrient-deficient vertebrate blood, which is compensated by the vitamin product(s) provided by *Wigglesworthia*. Tsetse reproduce viviparously, such that larvae mature within their mother’s sterile uterus and receive maternal milk gland secretions for nourishment. Two populations of *Wigglesworthia* exist in female tsetse: the first is intracellular in bacteriocytes which form the bacteriome organ in the anterior midgut, and the second is extracellular in the milk (Attardo, 2008, Balmand et al., 2013, Pais et al., 2008). *Wigglesworthia* in the adult bacteriome provide essential nutrients (including Vitamin B metabolites*)* that support the energy metabolism necessary for larval growth (Michalkova et al., 2014, Bing, 1857), while *Wigglesworthia* present free in milk secretions colonizes the gut bacteriome organ in the next generation of larvae (Balmand et al., 2013, Pais et al., 2008). Elimination of *Wigglesworthia* through dietary provisioning of antibiotics to mated females renders them unfecund, but fecundity can be restored in these flies by supplementing their diet with yeast extract and/or vitamins or with *Wigglesworthia* extracts obtained from dissected bacteriomes (Pais et al., 2008). This phenomenon has allowed us to maintain antibiotic-treated fertile lines that lack either only *Wigglesworthia* (*Gmm^Wgm-^*) (Pais et al., 2008), or that are completely symbiont-free (aposymbiotic; *Gmm^Apo^*) (Alam, 2011). Interestingly we noted that the adult progeny of tsetse that undergo larval development in the absence of *Wigglesworthia* in *Gmm^Wgm-^* and *Gmm^Apo^* present a severely compromised cellular immune system (Weiss et al., 2011), are highly susceptible to parasitism (Pais et al., 2008) and have a structurally compromised PM in their gut. We have observed that the adult *Gmm^Apo^* immune system lacks phagocytic hemocytes known as crystal cells (Wang, 2008), which can be restored when their mothers are fed a diet supplemented with *Wigglesworthia* cell extracts (Benoit, 2017). However, dietary supplementation with *Wigglesworthia* extracts fails to rescue the trypanosome susceptibility phenotype in the *Gmm^Apo^* adults, which present with a compromised PM. It remains to be shown what component of the obligate *Wigglesworthia* helps ensure the development of intact PM during the larval growth period (Weiss et al., 2013).

Fig. 1. Mammalian blood stream form trypanosomes enter into the gut lumen in an infected bloodmeal and differentiate to procyclic insect stage cells in the lumen shortly after acquisition. A thick peritrophic matrix (PM, Type II) originating from the cardia cells lines the entire midgut (MG) like a sleeve and becomes thicker after blood meals. Parasites bypass the PM barrier to colonize the Ectoperitrophic Space (ES) of the MG. Once detected by the epithelia, procyclic cells elicit epithelial immune responses which eliminate infections from the majority of flies although in a subset of flies parasites permanently colonize the ES and then move forward to colonize the anterior midgut and the cardia. In a subset of MG infected flies, parasites bypass the PM a second time to re-enter into the gut lumen and progress through the foregut. For transmission to the next mammalian host, parasites colonize the SG where they differentiate into short epimastigotes and eventually mammalian infective metacyclic forms, which are injected into the mammalian bite site in saliva. (Image contributed by Aurelien Vigneron, Aksoy lab).
